# Optical coherence tomography angiography findings in patients with Alport syndrome

**DOI:** 10.5935/0004-2749.20200088

**Published:** 2024-02-11

**Authors:** Flávio Gusmão Trancoso, Laisa Gallon, Maria Luiza de Azevedo Bomfim, Antônio Fábio Miguel da Silva, Fabiano Cade, Fernando Roberte Zanetti

**Affiliations:** 1 Department of Ophthalmology, Hospital Evangélico de Vila Velha, Vila Velha, ES, Brazil

**Keywords:** Retina, Tomography, optical coherence, Fluorescein angiography/methods, Nephritis, hereditary, Retina, Tomografia de coerência óptica, Angiofluoresceínografia/métodos, Nefrite hereditária

## Abstract

**Purpose:**

To describe the findings on optical coherence tomography angiography associated with Alport syndrome.

**Methods:**

Descriptive study from a referral ophthalmology service (Hospital Evangélico de Vila Velha, Brazil). Patients diagnosed with Alport syndrome were included.

**Results:**

The study group consisted of four patients (one female and three males) diagnosed with Alport syndrome. Visual acuity in the worst eye was between 20/40 and 20/60. All male patients had anterior lenticonus on biomicroscopy. The observed retinal findings included dots and flecks and pigmentary changes in the macula. On optical coherence tomography angiography, the inner retinal layers of all patients displayed thinning (especially in the temporal quadrant of the macula) and an increase in the foveal avascular zone. A thick choroid was observed in both eyes of the two youngest patients.

**Conclusions:**

In patients with Alport syndrome, the inner retinal layers suffer changes due to type IV collagen mutations. Optical coherence tomography angiography makes it possible to visualize and document these findings, making it a useful tool in the detection of early retinal findings associated with Alport syndrome.

## Abstract (pt)

**Objetivos:**

Descrever os achados na angiografia por tomografia de coerência óptica associada à síndrome de Alport.

**Métodos:**

Estudo descritivo de um serviço de referência em Oftalmologia (Hospital Evangélico de Vila Velha, Brasil). Os pacientes diagnosticados com síndrome de Alport, foram incluídos.

**Resultados:**

O grupo de estudo foi composto por quatro pacientes (um feminino e três homens) com diagnóstico de síndrome de Alport. A acuidade visual no pior olho estava entre 20/40 a 20/60. Todos os pacientes do sexo masculino apresentaram lenticone anterior à biomiscroscopia. Os achados da retina observados incluíram pontos e manchas e alterações pigmentares na mácula. Na angiotomografia de coerência óptica, as camadas internas da retina de todos os pacientes apresentaram afinamento (especialmente na região temporal da mácula) e aumento da zona avascular foveal. Uma coroide espessa foi observada em ambos os olhos dos dois pacientes mais jovens.

**Conclusões:**

Em pacientes com síndrome de Alport, as camadas internas da retina sofrem alterações devido à mutação do colágeno tipo IV. A angiotomografia de coerência óptica permite visualizar esses achados, tornando-o uma ferramenta útil na detecção de achados iniciais da retina associados à síndrome de Alport.

## INTRODUCTION

Affecting 1 in 10,000 individuals^(1)^, Alport syndrome (AS) is caused by mutations in *COL4A3, COL4A4*, and *COL4A5*-three of the genes responsible for type IV collagen synthesis. Mutations in COL4A5 are related to the X chromosome and correspond to the min form of heredity (85%)^(2^,^3)^, whereas mutations in *COL4A3* and *COL4A4* are related to the autosomal recessive pattern^(1^,^2)^.

Type IV collagen is present in the basement membrane of a number of tissues in the human body. The α3α4α5 chain is found in glomeruli, the organ of Corti, and several ocular structures, including the cornea, lens, and retina^(1^,^3)^. Mutations in this specific chain induce changes (nephropathy associated with hearing loss and ocular changes) referred to as AS.

Because of the presence of anomalous type IV collagen, ocular changes may occur in Descemet’s membrane, Bowman’s layer, the anterior lens capsule, the inner limiting membrane, and Bruch’s membrane^(3)^. The associated ophthalmological conditions include posterior polymorphous corneal dystrophy, recurrent corneal erosion, anterior lenticonus, macular atrophy, macular or peripheral dot-and-fleck retinopathy, and giant macular hole^(1^-^3)^.

Swept source optical coherence tomography angiography (SS OCT-A) makes it possible to visualize the retinal layers and evaluate the choroidal layers by observing erythrocyte motion in retinal blood vessels. So, far, few authors^(4)^ have used this technology to study AS. The present study provides a description of SS OCT-A findings in patients with AS.

## METHODS

This is a descriptive and analytical study of four patients with AS from the same family who were treated at the ophthalmology outpatient service of Hospital Evangélico de Vila Velha (Espírito Santo, Brazil) in 2018. Information was collected regarding clinical history, visual acuity, biomicroscopy, retinal mapping, SS OCT, and SS OCT-A (Topcon DRI OCT Triton Swept Source). Patient A was the sister of patient B and the mother of patients C and D. Three patients (B, C, and D) had renal biopsies (the gold standard test for AS).

## RESULTS

### Patient A

A 50-year-old woman was referred for evaluation of visual changes in both eyes (OU) at our ophthalmology outpatient service. The patient reported progressive loss of visual acuity over the preceding 3 years, especially in the right eye (OD), and dialysis-dependent chronic kidney disease (CKD), but she denied having diabetes or systemic arterial hypertension (SAH).

**Figure 1:**
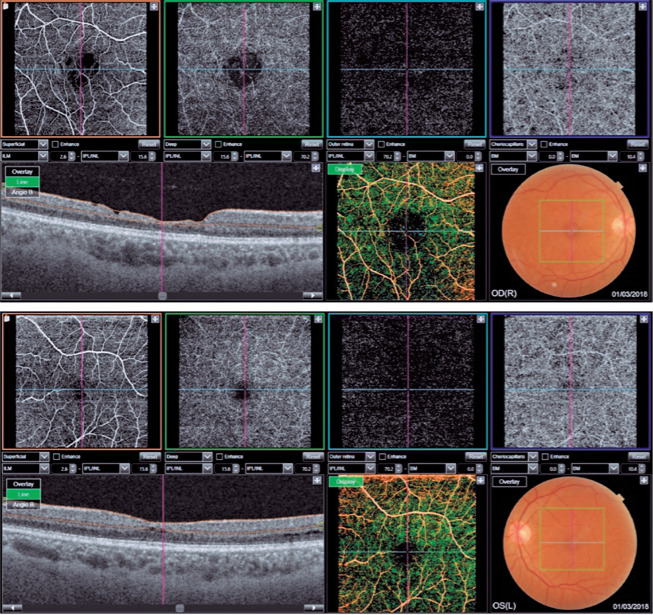
SS OCT scan of patient A. Atrophy of the inner retinal layers with “staircase” pattern, especially in the temporal quadrant of the macula in the right eye (OD), associated with increased foveal avascular zone (FAZ), and patchy flow voids in the choriocapillaris.

Upon examination, the best-corrected visual acuity was 20/60 OD and 20/30 in the left eye (OS). No significant findings were observed on biomicroscopy. Retinal mapping revealed pigmentary changes in the macula and dots and flecks in the posterior pole in OD but no changes in OS. On SS OCT, atrophy of the inner retinal layers with “staircase” pattern was observed, especially in the temporal quadrant of the macula in OD, and cysts were visible in the inner retinal layers in OS. On SS OCT-A, an increase was observed in the foveal avascular zone (FAZ) and in the number of patchy flow voids in the choriocapillaris (Figure 1).

### Patient B

A 35-year-old man with previously diagnosed AS, confirmed by renal biopsy, presented with SAH, hypoacusia, and dialysis-dependent CKD.

Upon examination, the best-corrected visual acuity was 20/25 OD and 20/60 OS. Biomicroscopy revealed a small anterior subcapsular cataract in OD and anterior lenticonus with pigments in the lens in OS. Retinal mapping showed pigmentary changes in the macula and parafoveal flecks in OU.

On SS OCT, atrophy of the inner retinal layers was observed in OD, especially in the temporal quadrant of the macula. SS OCT-A revealed increased and irregular FAZ. SS OCT and SS OCT-A could not be performed in OS due to poor gaze fixation.

### Patient C

A 28-year-old man with AS confirmed by renal biopsy presented with a history of SAH, hypoacusia, and non-dialysis-dependent CKD.

Upon examination, the best-corrected visual acuity was 20/40 in OU. Biomicroscopy revealed incipient lenticonus and pigments in the anterior lens capsule in OU. Retinal mapping showed macular pigmentary changes in OU.

**Figure 2:**
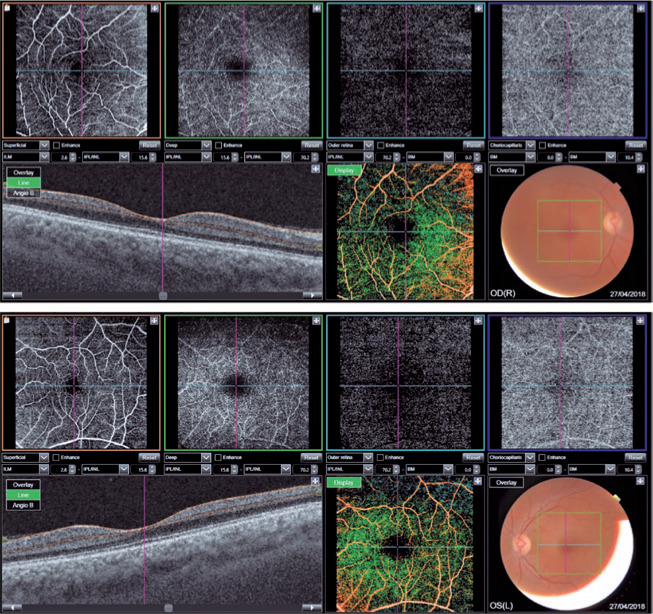
OCT-A scan of patient C showing atrophy of the inner retinal layers in both eyes (OU), especially in the temporal quadrant of the macula, associated with increased, and irregular foveal avascular zone (FAZ).

On SS OCT, atrophy of the inner retinal layers was observed in OU, especially in the temporal quadrant of the macula. On SS OCT-A, increased and irregular FAZ was seen in OU (Figure 2).

### Patient D

A 24-year-old man with AS confirmed by renal biopsy presented with SAH and non-dialysis-dependent CKD.

Visual acuity was 20/40 in OD and 20/30 in OS. Biomicroscopy revealed incipient lenticonus in OU. Retinal mapping showed macular pigmentary changes in OU.

**Figure 3:**
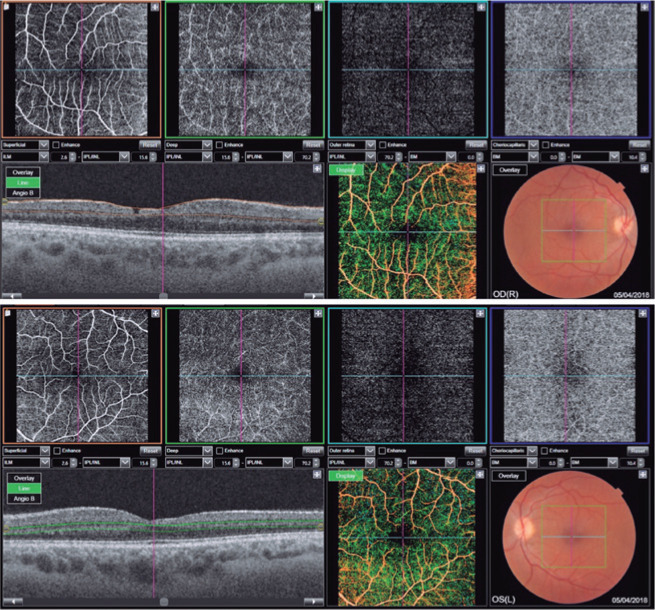
OCT-A scan of patient D showing atrophy of the inner retinal layers in both eyes (OU), especially in the temporal quadrant of the macula, associated with increased, and irregular foveal avascular zone (FAZ) in OU. A defect is also visible in the inner retinal layers in the right eye (OD).

On SS OCT, atrophy of the inner retinal layers was observed in OU, especially in the temporal quadrant of the macula, and defects were visible in the inner retinal layers in OD. SS OCT-A showed increased and irregular FAZ in OU (Figure 3).

## DISCUSSION

Lenticonus, a commonly observed ocular change in AS^(5)^, was detected in three of the four patients in our study. Macular or peripheral flecks^(2)^ were another frequent finding. Table 1 summarizes the findings observed in our study.

**Table 1:** Summary of changes in the OCT-A in patients with Alport syndrome

Patient	Age (yr)	Sex	Lenticonus	Retinal flecks	Retinal thinning	Changes in FAZ	Patchy flow voids
A	50	F	No	Yes	Yes	Yes	Yes
B	35	M	Yes	Yes	Yes	Yes	No
C	28	M	Yes	Yes	Yes	Yes	No
D	24	M	Yes	Yes	Yes	Yes	No

FAZ= foveal avascular zone.

One of the most prevalent findings, atrophy of the inner retinal layers, was found in all four patients (mostly early-stage disease), probably due to thinning of the inner limiting membrane and the nerve fiber layer^(4)^. Retinal thinning is more predominant in the temporal quadrant of the macula and in some cases may display a “staircase” pattern^(4)^, as shown by two of our patients (A and B).

According to Swaminathan et al.^(4)^, some AS patients develop intraretinal cysts near the macula. This was observed in one of our patients (A).

Our OCT-A findings matched the findings described by Swaminathan et al.^(4)^: increase of the FAZ and reduced density of the choriocapillary layer.

Most of the eyes of our patients displayed FAZ changes (increase and/or irregularity). These findings may be related to type IV collagen mutations causing atrophy of the inner retinal layers. The mutations can also induce changes in the microvasculature^(4)^.

Reduced density of the choriocapillary layer was only observed in the oldest patient of the series, suggesting it is a late change in AS. In contrast, FAZ changes are common in the early stages.

A thick choroid, the basis of the “pachychoroid” term, can be defined as a choroid with thickness >390 µm^(6)^. A thick choroid was observed in OU of the two younger patients, as shown in table 1.

No outer retinal damage was detected in our patients, not even in the patient with changes in the choriocapillary layer or in those with macular pigmentary changes.

To our knowledge, no other AS case series has been published describing the most prevalent findings observed on SS OCT-A.

## References

[1] Savige J, Sheth S, Leys A, Nicholson A, Mack HG, Colville D. (2015). Ocular features in Alport syndrome: pathogenesis and clinical
significance. Clin J Am Soc Nephrol.

[2] Igami TZ, LavezzoII MM, FerrazII DA, TakahashiI WY, Nakashima Y. (2012). Unusual macular thickness in Alport syndrome: Case
report. Arq Bras Oftalmol.

[3] Alves FR, Ribeiro FA. (2005). Revisão sobre a perda auditiva na Síndrome de
Alport, analisando os aspectos clínicos, genéticos e
biomoleculares. Rev Bras Otorrinolaringol.

[4] Swaminathan SS, Shah P, Zheng F, Gregori G, Rosenfeld PJ. (2018). Detection of choriocapillaris loss in Alport syndrome with
swept-source OCT angiography. Ophthalmic Surg Lasers Imaging Retina.

[5] Vedantham V, Rajagopal J, Ratnagiri PK. (2005). Bilateral simultaneous anterior and posterior lenticonus in
Alport’s syndrome. Indian J Ophthalmol.

[6] Lehmann M, Bousquet E, Beydoun T, Behar-Cohen F. (2015). PACHYCHOROID: an inherited condition?. Retina.

